# Interleukin-17 in Health and Disease: Special Focus on Its Role in Autoimmune Hepatitis

**DOI:** 10.3390/cells15100915

**Published:** 2026-05-17

**Authors:** Ştefan Agoston, Alina Grama, Alexia Onaciu, Alexandra Mititelu, Gabriel Benţa, Tudor Lucian Pop

**Affiliations:** 1 Faculty of Medicine, “Iuliu Hatieganu” University of Medicine and Pharmacy, 400012 Cluj-Napoca, Romania; agoston.stefan@elearn.umfcluj.ro (Ş.A.); onaciu.alexia@elearn.umfcluj.ro (A.O.); 2 2nd Pediatric Discipline, Department of Mother and Child, Faculty of Medicine, “Iuliu Hatieganu” University of Medicine and Pharmacy, 400012 Cluj-Napoca, Romania; perta.alexandra@elearn.umfcluj.ro (A.M.); benta.gabriel@elearn.umfcluj.ro (G.B.); tudor.pop@umfcluj.ro (T.L.P.); 3 2nd Pediatric Clinic, Emergency Clinical Hospital for Children, 400370 Cluj-Napoca, Romania

**Keywords:** interleukin-17, autoimmune hepatitis, inflammation, therapy

## Abstract

Autoimmune hepatitis (AIH) is a progressive inflammatory liver disease characterized by hypergammaglobulinemia, circulating antibodies, and distinctive histological features, with a higher prevalence in females. Immune responses targeting hepatic antigens are considered the main mechanism behind AIH. Many cytokines are involved in the inflammatory response typical of this disease. Interleukin 17 (IL-17) is a powerful pro-inflammatory protein that serves as a key link between the innate and adaptive immune systems. It plays an important role in regulating the inflammatory response in various tissues, including the liver. Several studies have shown that increased IL-17 levels are associated with the severity and progression of AIH. This review explores IL-17’s role in the AIH inflammatory pathway and summarizes existing evidence linking it to liver damage. We also highlight the potential of future therapies targeting this cytokine.

## 1. Introduction

The liver is a unique immunological organ. Its immune system initially responds to inflammatory stimuli through the activation of nonspecific immunity, primarily via natural killer (NK) and natural killer T (NKT) cells, followed by the activation of antigen-specific lymphocytes of the adaptive immune system. The development of autoimmune liver diseases is thought to result from an imbalance between pro-inflammatory immunity (which increases) and anti-inflammatory immunity (which decreases) [1].

Autoimmune liver diseases in children present with hepatic inflammation, elevated immunoglobulin G (IgG) levels, and specific autoantibodies. There are three medical conditions with a possible autoimmune origin: autoimmune hepatitis (AIH), autoimmune sclerosing cholangitis (ASC), and ‘de novo’ autoimmune hepatitis, which occurs following a liver transplant. AIH represents the prototype of autoimmune liver diseases. It is a progressive inflammatory liver disease characterized by hypergammaglobulinemia, circulating antibodies, and distinctive histological features, with a higher prevalence among females.

Autoimmune hepatitis (AIH) has traditionally been classified into two major subtypes: type 1 (AIH-1), characterized by antinuclear (ANA) and anti-smooth muscle antibodies (ASMA), and type 2 (AIH-2), associated with anti-liver-kidney microsomal type 1 (anti-LKM1) and anti–liver cytosol type 1 (anti-LC1) antibodies. The later identification of antibodies against soluble liver antigen/liver–pancreas (anti-SLA/LP) led to the proposal of a third subtype (AIH-3). However, large observational and cohort studies have demonstrated that disease severity, treatment response, and long-term prognosis are largely independent of autoantibody status, with substantial overlap in clinical, biochemical, and histological features across serological groups. Consequently, subclassification of AIH based solely on autoantibody profiles appears to have limited clinical utility, particularly in adults, and is no longer recommended. AIH responds in most cases to immunosuppressive treatment. Still, therapy must begin immediately after diagnosis, as untreated disease may lead to liver failure, a stage that in some cases may even require liver transplantation [2,3].

Smooth muscle antibodies lacking defined antigenic specificity are detected in both viral and non-viral liver diseases and are considered nonspecific serological markers of autoimmune hepatitis (AIH). Their antigenic targets are heterogeneous, including actin, tubulin, vimentin, and desmin. Among these, actin (particularly filamentous actin (F-actin) within cytoskeletal microfilaments) is recognized as the principal target in AIH type 1 [4].

The etiopathogenesis of AIH is a complex, multifactorial process involving interactions among environmental factors, immune system dysregulation, and individual genetic predisposition, including the major histocompatibility complex (MHC) and other genes involved in immune response regulation.

Environmental factors linked to genetic predispositions may trigger strong immune reactions, resulting in hepatocyte damage caused by autoantibodies. Viral infections (Epstein–Barr, varicella-zoster, hepatitis A, B, C, and E), lifestyle choices, and exposure to various xenobiotics are the major environmental factors that can initiate autoimmune hepatocyte damage. Exposures to toxins or drugs, such as nitrofurantoin, minocycline, or amoxicillin–clavulanate, or diets that affect the gut microbiome and increase intestinal barrier permeability, also contribute to the development of AIH [5,6,7,8].

Over the past few decades, the discovery of various T cell subsets has greatly advanced our understanding of the pathophysiology of inflammatory diseases. Effector T helper (Th) cells originate from naive CD4+ T cells upon activation through T cell receptor (TCR) engagement and co-stimulatory signals in the presence of specific cytokines. The major subsets of activated CD4+ T cells include specialized Th1, Th2, and regulatory T (Treg) cells, each possessing distinct functional roles. The identification of additional T cell subsets, such as Th17, Th9, Th22, and follicular Th cells, has enhanced our understanding of how the adaptive immune system contributes to inflammation and autoimmune processes. Among these subsets, Th17 cells play a particularly important role in hepatic autoimmunity, driving chronic inflammation by secreting pro-inflammatory cytokines such as IL-17 and IL-22 [6,7,8,9].

Genetic studies have identified cytotoxic T-lymphocyte–associated antigen 4 (CTLA-4, an adhesion molecule with a strong inhibitory role in the activation of these lymphocytes) as a potential non-MHC gene that predisposes to autoimmunity [10,11,12]. The Th cells, which are part of the body’s adaptive immune system, play a key role in initiating and maintaining hepatic inflammation in autoimmune liver diseases. They coordinate the immune response by secreting cytokines that regulate the activity of other immune cells, such as B lymphocytes, macrophages, and cytotoxic cells, thereby activating, proliferating, or inhibiting them. Pro-inflammatory cytokines (IL-1, IL-6, TNF-α) and anti-inflammatory cytokines (IL-4, IL-5, IL-6, IL-10) are linked by different subsets of Th cells, such as Th1 (IL-2, TNF-α, IFN-γ) and Th2 (IL-4, IL-5, IL-6, IL-10), thus influencing the balance between pro-inflammatory and anti-inflammatory responses and the dynamics of the immune process. An important role in this process is also played by Th17 cells, which induce and amplify the inflammatory response in the liver by secreting pro-inflammatory cytokines, such as IL-17 and IL-22. These stimulate the recruitment of neutrophils and other inflammatory cells, activate hepatic epithelial and endothelial cells, and amplify the local inflammatory response, thereby contributing to chronic liver inflammation and hepatocellular injury [13].

Susceptibility to AIH is influenced by genes in the human leukocyte antigen (HLA) region on the short arm of chromosome 6, especially those encoding DRB1 alleles. Genetic studies have identified CTLA-4 as a potential non-MHC gene linked to autoimmune liver diseases. These studies focus on the A+49G single-nucleotide polymorphism (SNP), located in exon 1 of the CTLA-4 gene, which involves replacing guanine with adenine at position 49 of the first exon. This change results in a switch from threonine to alanine at position 17 of the peptide. This modification affects the expression and cell-surface transport of CTLA-4, reducing its inhibitory effect on T-cell activation and thereby increasing the risk of autoimmune diseases such as AIH, type 1 diabetes, Graves’ disease, systemic lupus erythematosus, and rheumatoid arthritis [10,11,12]. Current evidence suggests that the major histocompatibility complex (MHC) plays a role in the initiation of idiopathic autoimmune hepatitis. Specifically, the alleles HLA-DRB103:01, DRB301:01, and DRB1*04:01, which encode HLA-DR3, DR52, and DR4 molecules, respectively, are strongly linked to susceptibility to type 1 AIH in European and North American populations, while their frequency is notably lower in Mediterranean populations, such as in Italy, compared to North American cohorts [5,14]. Furthermore, the impact of HLA-DR4 is highly context-dependent. While it is a major risk factor in North America, it lacks a significant association with AIH in certain other regions. However, HLA-DR4 remains a critical marker for disease onset in the elderly population, where it often correlates with a milder clinical phenotype and better treatment response [15,16]. Additionally, regional protective markers have been identified, most notably HLA-DR11, which serves as a distinctive protective factor against Type 1 AIH in Italian populations [14]. These findings underscore how local genetic backgrounds shape the global diversity of AIH clinical presentations.

This narrative review aims to describe IL-17’s role in the development of autoimmunity, summarize data on its involvement in the liver’s inflammatory response in AIH, and analyze it as a potential therapeutic target.

## 2. IL-17 Family and IL-17 Receptors

The effector cytokines of Th17 include IL-17, IL-21 (which promotes self-activation of Th17 and B cells, and immunoglobulin production), and IL-22 (which protects against liver injury) [12,13,17,18]. IL-17 is produced by the Th17 cell subset, which also secretes IL-17F, IL-21, IL-22, IL-6, and TNF-α [18,19]. This cytokine is expressed by CD4+ T cells, CD8+ T cells, NKT cells, and Treg cells. The differentiation factors for Th17 cells include transcription factors (STAT3, IRF4, RORα, RORγt), cytokines (TGFβ, IL-6/IL-21), and the maturation factor IL-23 [1,7,12,13,17,18]. As a key pro-inflammatory cytokine, IL-17 plays a crucial role in regulating inflammation and is influenced by the host’s health status. Under normal physiological conditions, IL-17 production remains low and stable. In healthy individuals, TGF-β suppresses Th17 cell activation, preventing excessive IL-17 secretion and maintaining immune tolerance. However, during pathogen invasion, Th17 cell activation is increased by the combined influence of TGF-β and IL-6, leading to elevated IL-17 secretion and promoting inflammation. Dysregulation of IL-17 production can result in autoimmune disorders and tissue damage, with excessive IL-17 levels associated with the onset and progression of various autoimmune diseases [13,19].

The first member of the IL-17 cytokine family to be identified was IL-17A, but searches for similar genes led to the discovery of IL-17B, IL-17C, IL-17D, IL-17E (now called IL-25), and IL-17F. These cytokines have an unusual cystine-knot fold structure, like nerve growth factor (NGF) and platelet-derived growth factor (PDGF), but distinct from other immune cytokines [19,20,21].

**Figure 1:**
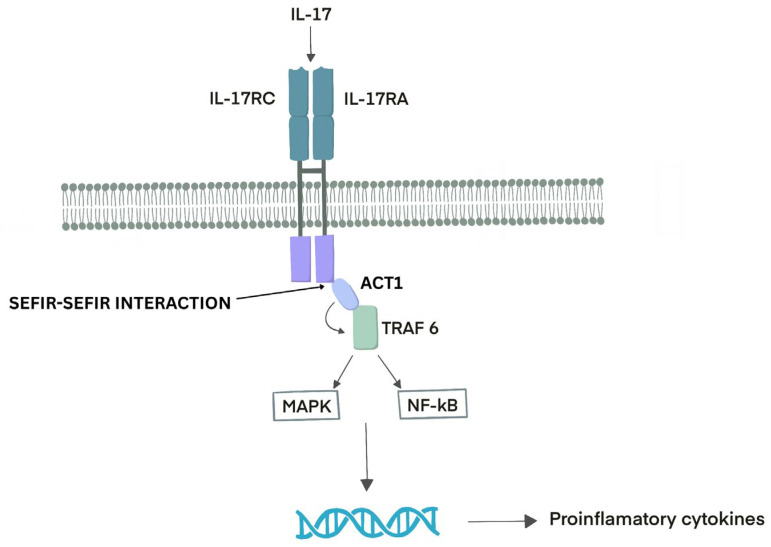
IL-17 Receptor Signaling Pathway. The IL-17 receptor (IL-17R) is composed of two widely expressed subunits, IL-17RA and IL-17RC. The binding of dimeric IL-17 induces receptor heterodimerization and recruitment of the cytoplasmic adaptor protein ACT1, promoting the expression of pro-inflammatory cytokine genes through mRNA stabilization and activation of downstream signaling pathways. These pathways involve recruitment of tumor necrosis factor receptor-associated factor (TRAF) E3 ubiquitin ligases, particularly TRAF6, which generates K63-linked polyubiquitin chains that serve as docking platforms for signaling complexes, ultimately activating the mitogen-activated protein kinase (MAPK) and nuclear factor-κB (NF-κB) pathways and driving pro-inflammatory cytokine production.

As a unique cytokine receptor family, the IL-17 receptor (IL-17R) subunits have a conserved cytoplasmic motif, the SEFIR domain, characteristic of the IL-17R family and functionally analogous to the Toll/IL-1 receptor (TIR) domain present in Toll-like receptors (TLRs) and IL-1 receptors [21]. The IL-17R consists of two broadly expressed subunits, IL-17RA and IL-17RC. When dimeric IL-17 binds to the receptor, it triggers heterodimerization and the recruitment of the cytoplasmic protein ACT1. This recruitment enhances the expression of genes encoding pro-inflammatory cytokines by stabilizing their mRNAs or activating downstream signaling pathways that promote their transcription. These gene-activation pathways rely on the recruitment of E3 ubiquitin ligases from the tumor necrosis factor (TNF) receptor-associated factor (TRAF) family, particularly TRAF6. TRAF6 generates non-degradative K63-linked polyubiquitin chains, which act as docking platforms for various signaling molecules and facilitate the activation of downstream pathways, most notably the mitogen-activated protein kinase (MAPK) and nuclear factor-κB (NF-κB) cascades, leading to the production of pro-inflammatory cytokines. Interestingly, IL-17-induced signaling is relatively weak compared to that triggered by other pro-inflammatory cytokines such as IL-1α or TNF, even though their receptors all utilize non-degradative polyubiquitin linkages and share several proximal signaling components [22] [Figure 1].

IL-17A (often referred to simply as IL-17) is the most widely studied member of the IL-17 cytokine family [7]. The human IL-17A monomer is a glycoprotein composed of 155 amino acids. During the synthesis of the 35 kDa homodimer for expression, the N-terminal 23-amino acid signal peptide is cleaved, and a disulfide bond is formed. Within the IL-17 family, IL-17F (produced by Th cells) shares the greatest similarity to IL-17A, with approximately 55% sequence homology [20].

IL-17B has been reported to exert anti-inflammatory effects by inhibiting IL-25-mediated signaling during mucosal inflammation, a mechanism attributed to their shared utilization of the IL-17RB receptor subunit [23]. Comparable to IL-17, IL-17C contributes to antimicrobial defense mechanisms and supports the maintenance of epithelial barrier integrity in the skin and intestine [24,25]. IL-17D remains the most poorly characterized member of the IL-17 cytokine family. It is broadly expressed across various healthy tissues and is elevated in tumor contexts and during viral infections [21]. IL-17E (also known as IL-25) represents an atypical member of the IL-17 cytokine family. While most IL-17 family cytokines are known to promote neutrophilic inflammation, IL-25 uniquely stimulates the production of IL-4, IL-5, IL-13, and thymic stromal lymphopoietin-cytokines characteristically associated with type 2 immune responses. Owing to this distinct functional profile, IL-25 was assigned a separate interleukin designation to emphasize its divergence from other IL-17 family members [26].

IL-17F exhibits the greatest structural and functional similarity to IL-17A, particularly regarding their cellular sources and biological roles. The genes encoding IL-17A and IL-17F are co-located and are typically co-expressed by type 17 cells. Both IL-17A and IL-17F form homodimers, although they can also assemble into an IL-17A/F heterodimer. These cytokine variants signal through a shared receptor complex composed of the IL-17RA and IL-17RC heterodimer. Given their shared receptor components, IL-17A, IL-17A/F, and IL-17F activate qualitatively similar downstream pathways. Nevertheless, IL-17A homodimers elicit a substantially stronger signaling response compared to IL-17F homodimers, while the IL-17A/F heterodimer mediates an intermediate level of signaling activity [21].

A complex network of interacting factors governs the regulation of pathological versus protective IL-17 responses. Key transcription factors, such as RORγt, STAT3, IRF4, and FoxP3, are central to the differentiation, function, and balance between Th17 cells and Tregs, ultimately determining the outcome of IL-17–driven immune responses. RORγt, the master transcription factor of IL-17 expression, directs naive CD4^+^ T cells to differentiate into Th17 cells that secrete IL-17. STAT3 activation further enhances IL-17 production by promoting its transcription, while IRF4 cooperates with RORγt by binding to the IL-17 gene promoter to modulate its expression. In contrast, FoxP3 is essential for the development and suppressive function of regulatory T cells [3,5,6,7,8,9]. Disruption in the regulation of these transcription factors can lead to excessive IL-17 production or defective immune regulation, contributing to the pathogenesis of inflammatory diseases [7,8,9]. While transient and regulated IL-17 expression supports physiological processes that promote host immune defense and tissue repair, sustained or chronic IL-17 activation drives pathogenic responses that facilitate the development of autoimmune disorders and cancer [20]. A deeper, more comprehensive understanding of the mechanisms underlying IL-17 signal transduction is essential to enhance the efficacy of IL-17-targeted therapies for inflammatory and autoimmune diseases and to enable more precise therapeutic interventions in cancer [20].

The IL-17R signaling cascade is initiated by the recruitment of CT1 (also known as CIKS), a multifunctional adaptor protein that contains an SEFIR domain essential for its interaction with IL-17R. ACT1 functions as a nonredundant mediator, playing a critical role in activating IL-17RA-dependent signaling pathways [18,24]. IL-17 can promote mRNA stabilization through the adaptor protein CT1 and the signaling molecules TRAF2 and TRAF5. These factors regulate mRNA stability either directly or indirectly by influencing the activity of mRNA-binding proteins such as ARID5A and HuR, the splicing factor SF2, and the endoribonuclease Regnase-1 [22].

Following IL-17 stimulation, Act1 rapidly associates with and ubiquitinates TRAF6, an E3 ubiquitin ligase. Similar to other receptors that use TRAF6, engagement of IL-17 activates the canonical nuclear factor κB (NF-κB) pathway by stimulating IκB kinase and leading to the degradation of IκBα. Activated NF-κB then induces the transcription of IκBζ and B-cell lymphoma 3-encoded protein (Bcl3), noncanonical NF-κB family members that, in turn, facilitate the expression of a broad range of IL-17– and NF-κB–dependent pro-inflammatory and antimicrobial genes [21].

## 3. Different Immune Roles of IL-17

The role of IL-17 in hepatic autoimmunity remains unclear. Several studies have demonstrated that increased IL-17 expression is associated with the severity and progression of AIH, suggesting that this cytokine contributes to the maintenance of hepatic inflammation and tissue injury.

The pathogenic role of Th17 cells, as a marker of autoimmunity, has also been demonstrated in various diseases, including rheumatoid arthritis, type 1 diabetes, Sjögren’s syndrome, thrombocytopenia, multiple sclerosis, and autoimmune thyroid diseases. IL-17 also has a well-established role in the pathology of psoriasis, psoriatic arthritis, and ankylosing spondylitis. Th17 cells are found in the dermis of psoriasis skin lesions and mediate skin inflammation in mice and humans following recognition of self-lipid antigens presented by CD1a. Furthermore, IL-17-producing CD8+ T cells with a tissue-resident phenotype are found in the synovial fluid of patients with psoriatic arthritis [27].

IL-17 plays a pivotal role in host defense mechanisms against mucosal infections. Furthermore, it serves as a major pathogenic mediator and therapeutic target in various autoimmune and inflammatory disorders, as well as malignancies. Beyond its involvement in inflammatory responses, IL-17 also participates in a broad range of physiological and pathological processes in vivo [20]. The expression levels of IL-17 are markedly elevated in both the serum and inflamed mucosal tissues of individuals with active ulcerative colitis or Crohn’s disease. IL-17, primarily secreted by Th17 cells and/or innate lymphoid cells (ILCs), plays a pivotal role in the pathogenesis of intestinal inflammation by activating the IL-23/IL-23R signaling pathway. Evidence from murine colitis models indicates that IL-23 promotes IL-17 production, thereby amplifying and sustaining localized intestinal inflammation [28].

A key feature of IL-17 signaling is its strong dependence on cooperative interactions with other cytokines to elicit effective responses. On its own, IL-17 serves as a relatively weak inducer of signaling cascades and downstream gene expression. Rather, it exerts its functional impact through synergistic interactions with a broad range of cytokines, including classical pro-inflammatory mediators such as tumor necrosis factor (TNF) and interferon gamma (IFN-γ), as well as the ostensibly anti-inflammatory transforming growth factor beta (TGF-β). Additionally, IL-17 can potentiate lipopolysaccharide (LPS)-induced signaling via Toll-like receptor 4 (TLR4). In many cases, however, the molecular mechanisms underlying these synergistic interactions remain incompletely understood [29].

IL-17 is also produced by other immune cell populations, including CD8^+^ cytotoxic T (Tc17) cells, γδ T cells, NKT cells, group 3 innate lymphoid cells (ILC3s), and “natural” Th17 cells. [18]. Therefore, elucidating the interaction between IL-17 and its downstream metabolic pathways may provide valuable insights for the development of therapeutic strategies targeting IL-17–associated diseases, including those not conventionally linked to this cytokine [27].

During the immune response to infections, IL-17 expression is typically transient. In contrast, sustained IL-17 production is observed in many chronic inflammatory and autoimmune disorders, where its prolonged activity may significantly contribute to the development or exacerbation of metabolic syndrome [27].

The responsiveness of IL-17 target cells is primarily attributed to the limited expression of the critical IL-17RC subunit. In contrast to Th17 cells differentiated in vitro, IL-17RC expression is typically low or undetectable in hematopoietic cells. Given that IL-17–responsive cells are distributed across a wide range of tissues and organs, it is unsurprising that each cell type exhibits unique metabolic profiles and nutrient utilization patterns [27].

IL-17A mainly acts on non-hematopoietic cells, particularly epithelial cells, and consistently participates in protective immunity at boundary tissues. Regarding the skin, IL-17A promotes keratinocyte proliferation and aberrant differentiation and contributes to skin barrier disruption by downregulating the expression of molecules involved in keratinocyte differentiation, such as filaggrin [30]. One hallmark function of IL-17 is the induction of chemokines, including CXCL1, CXCL2, and CXCL8 (IL-8), that attract myeloid cells like neutrophils to the infected or injured tissue [31].

IL-17 serves as a critical mediator of barrier tissue repair. Evidence for IL-17’s regenerative function in the gastrointestinal tract emerged from observations in patients with inflammatory bowel disease, in whom treatment with anti–IL-17A or anti–IL-17RA biologics unexpectedly exacerbated symptoms during clinical trials for Crohn’s disease. In the skin, IL-17 facilitates wound healing, and studies in murine models have shown that impaired IL-17 signaling delays wound closure, compromises barrier integrity, and alters the skin microbiota. While IL-17–mediated signaling supports cellular proliferation and tissue regeneration, its dysregulation can contribute to tumorigenic processes [27]. The effects of IL-17A are not limited to keratinocytes; they also affect endothelial cells, fibroblasts, chondrocytes, and synovial cells. IL-17A is clearly of major importance in the context of psoriasis-associated comorbidities, namely psoriatic arthritis and cardiovascular disease/atherosclerosis [30].

IL-17 also plays a role in maintaining glucose metabolic homeostasis. In vitro studies have shown that IL-17 suppresses insulin-stimulated glucose uptake in adipocytes. In vivo, young IL-17-deficient mice exhibit mildly elevated fasting glucose levels, reduced insulin levels, and enhanced glucose clearance during glucose tolerance testing. This effect diminishes with the onset of age-related obesity [27].

Adaptive thermogenesis is the physiological process of heat generation, primarily in brown adipose tissue. Emerging evidence indicates that IL-17 plays a role in regulating this process. Notably, recent studies have demonstrated that IL-17F, rather than IL-17A, influences thermogenic activity by enhancing TGF-β1 expression in adipocytes, thereby promoting sympathetic innervation of adipose tissue. This neural stimulation facilitates lipolysis and thermogenesis through norepinephrine signaling [27].

## 4. Role of IL-17 in Infectious Diseases

Significant roles of IL-17 signaling have been identified in host defense against a range of viral and intracellular bacterial infections. In murine models, IL-17 has been shown to enhance cytotoxic T cell activity against West Nile virus and to facilitate the recruitment of CD8^+^ cytotoxic T cells to the liver during acute hepatitis [29].

The coronavirus disease 2019 (COVID-19) pandemic has vividly demonstrated the potentially fatal consequences of an excessive cytokine response. Approximately 10–20% of confirmed cases progress to a stage requiring hospitalization and oxygen therapy during the second phase of infection, particularly when the virus induces acute respiratory distress syndrome. IL-17 and its downstream mediator IL-6 have been involved as key contributors to this immunopathology. Notably, a clinical trial in China is investigating the therapeutic potential of ixekizumab, an IL-17A-neutralizing antibody originally developed for psoriasis, in the treatment of severe acute respiratory syndrome coronavirus 2 (SARS-CoV-2) infection [29].

Patients with chronic viral hepatitis are at increased risk for developing progressive fibrosis, cirrhosis, and hepatocellular carcinoma. Elevated intrahepatic levels of IL-17A and IL-22 detected at the time of biopsy are regarded as biomarkers of advanced fibrosis and are associated with poorer clinical outcomes. Hepatic stellate cells are the principal mediators of liver fibrogenesis, and IL-17 has been shown to promote collagen production by these cells, in part by upregulating IL-17, which augmented the expression of genes encoding collagen and fibronectin in lymph node stromal cells that had been preactivated in vivo by immunization, and additionally facilitated the proliferation of these stromal cells [29].

Detection of IL-23 and IL-23R expression in liver biopsy samples from HBV-infected individuals has provided evidence of Th17 cell involvement in HBV infection. Moreover, IL-17 appears to be essential for the differentiation of naive CD4^+^ T cells into Th17 cells following HBsAg stimulation. Collectively, these findings indicate that Th17 cells contribute to the pathogenesis of HBV-associated liver injury [32].

The role of Th17 cells in hepatitis C virus (HCV) infection and disease progression remains incompletely understood. Studies have indicated that antigen-specific Th17 cells are generated in individuals infected with HCV. Additionally, TGF-β and IL-10, cytokines upregulated by the viral nonstructural protein 4, have been shown to suppress Th17 responses in HCV-infected patients. Notably, elevated levels of IL-17 have been observed in these patients compared with healthy controls, although no association with viremia has been established [32].

Patients with autoimmune hepatitis (AIH) may develop acute-on-chronic liver failure (ACLF), either as a hyperacute presentation of previously undiagnosed or misdiagnosed disease, or following a secondary exogenous insult (viral, drug-induced, or toxic), potentially facilitated by prolonged immunosuppression. Data on AIH-related cirrhosis remain heterogeneous, with some studies reporting survival outcomes comparable to non-cirrhotic patients, while others describe poor prognoses that may delay or preclude liver transplantation. Although reports of ACLF in AIH are limited, a Chinese cohort study of autoimmune liver disease (40% AIH) demonstrated high 28- and 90-day mortality rates (~40% and ~75%, respectively). Notably, 76% of patients had documented bacterial infections prior to admission, most commonly involving the respiratory tract (42.5%) and peritoneum (22.5%). In contrast, Anand et al. identified no precipitating factor in over 80% of cases, whereas Sharma et al. reported that more than half of cases were triggered by tapering of immunosuppressive therapy [33].

## 5. Role of IL-17 in Autoimmune Disorders

Skin inflammation. The role of IL-17 in the pathogenesis of psoriasis is well established. Chemokine-like MARVEL transmembrane domain-containing protein 4 (CMTM4), a component of the IL-17 receptor complex, contributes to the mediation of psoriatic inflammation. In both murine models and human subjects, Th17 cells are localized within the dermis of psoriatic lesions, where they regulate cutaneous inflammation in response to self-lipid antigens presented by the cluster of differentiation 1a molecule. Furthermore, IL-17 activity serves as a mechanistic link between psoriasis and metabolic-associated fatty liver disease. IL-17E, also known as IL-25, is abundantly expressed in lesional skin of individuals with psoriasis and, in a murine imiquimod-induced psoriasis model, has been shown to act through IL-17-mediated pathways. Exogenous IL-17E administration induces skin inflammation, whereas germline or keratinocyte-specific deletion of IL-17E confers increased resistance to imiquimod-induced psoriasis [20].

Inflammatory bowel disease (IBD). Although the underlying mechanisms remain incompletely understood, a significant genetic predisposition is evident. Beyond the classical Th1 and Th2 immune responses, additional T-cell subsets, particularly Th17 and Treg cells, are thought to contribute to the pathogenesis of IBD. This is supported by evidence suggesting that the IL-13/Th17 signaling axis is a potential biomarker of active disease, and that the presence of IBD, rather than genetic burden, influences the mRNA expression of IBD-associated Th17/IL-13 genes. Furthermore, elevated levels of circulating Th17 and Treg cells have been documented in the peripheral blood of individuals with IBD [32]. IL-17 levels are markedly elevated in the circulation and inflamed mucosal tissues of individuals with active ulcerative colitis or Crohn’s disease. Moreover, genome-wide association studies have identified a nonsynonymous polymorphism in IL23R that is associated with susceptibility to Crohn’s disease. IL-17, produced by Th17 cells and/or innate lymphoid cells and induced by IL-1 and IL-23, has been implicated in the development of chronic intestinal inflammation in murine colitis models. IL-23 further enhances IFN-γ production, which cooperates with IL-17 to drive intestinal inflammatory responses. This pathogenic mechanism may also involve ex-Th17 cells, a population of former Th17 cells that transition into IFN-γ–producing cells. In contrast, clinical studies in patients with IBD have shown that pharmacological blockade of IL-17 increases the risk of intestinal mucosal *Candida albicans* infection. Although IL-17 and Th17 cells can promote gut inflammation, IL-17 and IL-22 also exert protective effects against fungal and bacterial infections. Consistent with this dual role, treatment with IL-17 inhibitors has been associated with both new-onset IBD and exacerbation of pre-existing colitis [20].

Systemic lupus erythematosus (SLE). Evidence for the involvement of Th17 cells in SLE includes elevated serum IL-17 levels and increased circulating Th17 cells; however, studies have not demonstrated significant differences between patients with active versus inactive disease [32]. Multiple studies have reported dysregulated IL-17 levels in patients with SLE, with IL-17 concentrations in both plasma and affected tissues positively associated with disease severity. Notably, in two lupus animal models, suppression of IL-17 expression markedly changed SLE-related pathological manifestations. Despite these observations, the precise relationship between IL-17 levels and SLE severity remains inconclusive. Although macro-level evidence indicates that IL-17 levels fluctuate in parallel with clinical symptoms in both human patients and murine models, subsequent investigations have not demonstrated a consistent direct correlation. A later meta-analysis confirmed a positive association between IL-17 levels and SLE progression; however, the relatively weak correlation has prompted continued debate about the fundamental role of IL-17 in SLE pathogenesis [20].

Hashimoto’s thyroiditis (HT). Previous studies have demonstrated that both intrathyroidal Th17 cell infiltration and IL-17 serum levels are markedly elevated in patients with Hashimoto’s thyroiditis. Additionally, exposure to moderately high iodine levels has been shown to promote the differentiation of murine splenic naive T cells into Th17 cells, while excessively high iodine concentrations preferentially induce Th1 polarization and suppress Treg development. These findings suggest that Th1 and Th17 responses may contribute to the immunopathogenesis of HT and that elevated iodine intake may play a pivotal role by modulating T-cell differentiation profiles [34].

Multiple Sclerosis (MS). Several research groups have studied and characterized T-cell subsets and their cytokine profiles in MS, showing that both the frequency of Th17 cells and the levels of Th17-related cytokines are higher in people with MS than in healthy controls. Additionally, a lower Treg/Th17 ratio, along with a link between this ratio and disease severity, has been observed, suggesting a possible role in disease progression [28].

Spondyloarthritis. Regarding the IL-23/IL-17 axis in the pathogenesis of spondyloarthritis, elevated serum levels of IL-17 and IL-23 have been reported, along with an increased frequency of circulating Th17 cells [32].

Th17 cells play a vital role in the development of acute pancreatitis by releasing IL-17, involved in defending the host against extracellular bacteria and fungi. When activated by various cytokines, Th17 cells produce IL-21, which is crucial for the expression of RORγt and IL-17. This signaling pathway encourages T-cell proliferation while reducing Treg-mediated suppression [18,35].

Neoplasms. Beyond its role in autoimmunity, dysregulated IL-17 expression is a critical contributor to both early and advanced stages of human tumorigenesis. Variations in IL-17 levels significantly influence tumor initiation and progression across multiple organs, including the colon, liver, pancreas, lungs, and biliary tract. Moreover, evidence from preclinical models and clinical studies indicates that elevated serum IL-17 levels correlate with poor prognosis and reduced responsiveness to radiotherapy in patients with various solid tumors. These findings suggest that targeting IL-17 may suppress tumor metastasis and improve the radiotherapeutic sensitivity of cancer cells [20].

## 6. Evidence of the Role of IL-17 in AIH

Immune responses targeting hepatic self-antigens are considered the main mechanism behind AIH [36]. The immunological pathways that activate naive T cells and lead to autoimmune hepatocyte injury in AIH begin when antigen-presenting cells (APCs) present autoantigenic peptides to naive T cells. This activation requires proper co-stimulatory signals, especially through CD28 on naive T helper cells (Th0) interacting with CD80 on APCs. Self-antigenic peptides are processed and presented by specialized APCs, including dendritic cells (DCs), macrophages, and B lymphocytes. Importantly, this antigen presentation also occurs in the liver, which contains multiple APC populations, including Kupffer cells, liver sinusoidal endothelial cells (LSECs), DCs, hepatic stellate cells, and hepatocytes. In this hepatic environment, antigen presentation to both CD4^+^ and CD8^+^ effector T cells can happen locally, potentially bypassing the need for migration to regional lymphoid organs [37].

**Figure 2:**
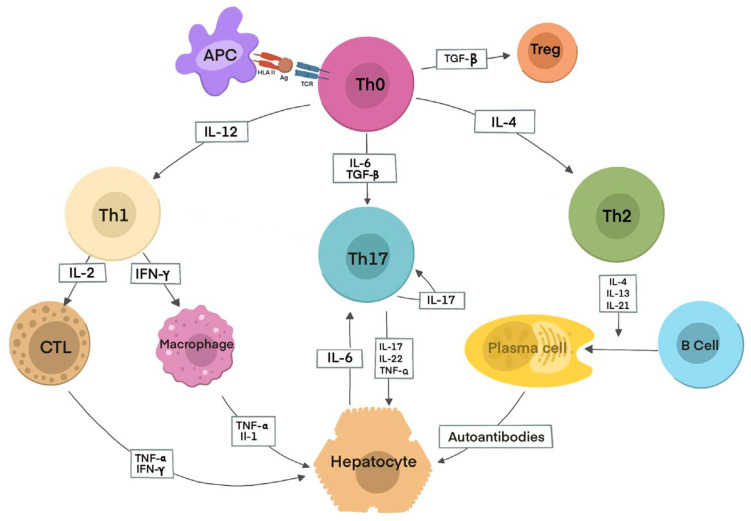
Autoimmune hepatitis inflammation pathways (adapted after Terziroli Beretta-Piccoli B. et al. [32]). Initially, an autoantigenic peptide is presented to the TCR of naïve Th0 lymphocytes by HLA class II molecules on antigen-presenting cells (APCs) in regional lymph nodes or the liver. Following activation, Th0 cells differentiate into Th1 or Th2 subsets in response to IL-12 or IL-4, respectively, depending on the antigenic stimulus. Th1 cells produce IL-2 and IFN-γ, activating cytotoxic T lymphocytes, upregulating HLA class I expression, inducing HLA class II on hepatocytes, and stimulating macrophages to release IL-1 and TNF-α. Th2 cells secrete IL-4, IL-13, and IL-21, promoting B-cell differentiation and autoantibody production. Regulatory T cells (Tregs) are induced by TGF-β, and their dysfunction permits hepatocyte injury via CTLs, pro-inflammatory cytokines, complement activation, and NK cell–mediated cytotoxicity. In the presence of TGF-β and IL-6, Th0 cells may also differentiate into Th17 cells, which produce IL-17, IL-22, and TNF-α; hepatocyte-derived IL-6 further enhances this inflammatory response.

Initially, an autoantigenic peptide is presented to the TCR of naive Th0 lymphocytes via HLA class II molecules on an APC, either in regional lymph nodes or directly in the liver. Upon activation, Th0 cells differentiate into Th1 or Th2 subsets in response to IL-12 or IL-4, respectively, depending on the antigenic stimulus. This differentiation triggers a cascade of immune responses determined by the cytokines produced. Th1 cells secrete IL-2 and IFN-γ, which activate cytotoxic T lymphocytes (CTLs), increase HLA class I expression, induce HLA II expression on hepatocytes, and activate macrophages, which release IL-1 and TNF-α. Th2 cells mainly produce IL-4, IL-13, and IL-21, promoting B lymphocyte differentiation into plasma cells and stimulating autoantibody production. Tregs differentiate from Th0 cells under the influence of TGF-β, and their deficiency or dysfunction facilitates hepatocyte destruction through effector mechanisms, including CTL activity, cytokines from Th1 cells and macrophages, complement activation, and NK cell adhesion to autoantibody-coated hepatocytes via Fc receptors. Th17 cells, arising from Th0 cells in the presence of TGF-β and IL-6, secrete pro-inflammatory cytokines such as IL-17, IL-22, and TNF-α. Hepatocytes also contribute to Th17 differentiation by releasing IL-6, thereby amplifying local inflammation [37] [Figure 2].

Dysfunctional regulatory circuits in Tregs may cause abnormal regulation of IL-17-driven inflammatory responses. Among Tregs, CD39^+^ Tregs have been shown to selectively suppress IL-17 immunity compared to their CD39^−^ counterparts. Recent evidence indicates that in AIH patients, CD39^+^ Tregs are not only decrease in number but also exhibit impaired ATP/ADP hydrolysis, reducing their ability to control excessive IL-17 responses by effector CD4^+^ T cells. These findings suggest that dysfunction of CD39^+^ Tregs in AIH, including their diminished capacity to inhibit IL-17 production and their tendency to differentiate into IL-17–producing effector cells, may contribute to defective immunosuppression and the development of AIH [38].

The inflammatory infiltrate in AIH mainly consists of α/β T lymphocytes, with CD4^+^ T cells roughly twice as common as CD8^+^ T cells. These findings were reported almost thirty years ago; therefore, it is timely to re-examine hepatic infiltrates in AIH using current insights and technological progress [37].

Haijing Yu et al. examined the role of IL-17 in the pathogenesis of both human and experimental AIH. Elevated IL-17 mRNA levels in the peripheral blood mononuclear cells of AIH patients, along with hepatic infiltration by IL-17-positive cells in the animal models, strongly suggest that the IL-17 pathway plays a central role in AIH development. Furthermore, treatment with a monoclonal antibody targeting IL-17A significantly reduced hepatic necrosis compared to control groups [39].

Li Zhao et al. explored the role of Th17 cells in the development of AIH. Serum IL-17 levels and the number of IL-17–positive cells in hepatic inflammatory infiltrates were significantly higher in patients with AIH than in healthy controls and those with chronic HBV infection. Additionally, IL-17 was found to increase IL-6 production by hepatocytes, which further activates Th17 cells. This creates a positive feedback loop between Th17 cells and hepatocytes, thereby intensifying the inflammatory response [36].

Gutkowski et al. found that IL-6 and IL-17 levels were significantly higher in patients with active AIH than in those in remission or in the control group. Conversely, serum IL-6 and IL-17 levels did not differ significantly between patients in remission and healthy controls. Additionally, a negative correlation was observed between the severity of inflammatory lesions and serum IL-17 levels [40].

Also, An et al. found that serum levels of IL-17, IL-6, IL-21, and TNF-α were significantly higher in patients with AIH compared to the control group. Additionally, serum IL-17 and TNF-α levels, as well as the frequency of Th17 cells, were positively correlated with serum transaminase levels [41].

Other studies have examined the role of IL-17 in the hepatic inflammatory response in AIH (Table 1). IL-17 serum levels are significantly higher in patients with AIH than in controls, with some studies suggesting differences between mild and severe cases [42,43,44,45,46,47,48,49]. Similar findings were seen in experimental studies using murine models [38,43,49,50,51,52]. Some researchers also used increased IL-17 expression in hepatic tissues as an indicator [43,46,50,52]. It has been proposed that after treatment with anti-IL-17 antibodies, liver inflammation and related indicators decreased [43,50,52].

## 7. Possible Therapies Involving IL-17

Among the available strategies for targeting the IL-17 signaling pathway, blocking IL-17 cytokines or their receptors is the most direct therapeutic approach. Secukinumab is a recombinant human IgG1/kappa monoclonal antibody that selectively targets IL-17A, thereby preventing its binding to the IL-17 receptor (IL-17R) and subsequently inhibiting the production of downstream pro-inflammatory cytokines and chemokines involved in disease development. In contrast, whereas secukinumab specifically neutralizes IL-17A, brodalumab acts at the receptor level by blocking IL-17R, thus inhibiting signaling mediated by multiple IL-17 cytokines. Ixekizumab is a humanized IgG4 monoclonal antibody that also selectively binds IL-17A, preventing its interaction with IL-17R and suppressing the downstream secretion of pro-inflammatory cytokines and chemokines from target cells. Preclinical studies have shown that ixekizumab and bimekizumab (the first dual inhibitor of IL-17A and IL-17F) exhibit comparable or higher binding affinity for IL-17A than secukinumab, while bimekizumab uniquely demonstrates potent neutralization of IL-17F [51].

Therapeutic strategies targeting pathways upstream of IL-17 signaling have emerged as promising areas of research. Several proinflammatory cytokines, especially IL-23, IL-6, and IL-1β, play crucial roles in the differentiation and expansion of IL-17–producing cells, thereby promoting IL-17 expression. Monoclonal antibodies that target the IL-6 receptor, such as tocilizumab, have been approved in many countries for the treatment of inflammatory autoimmune diseases, including rheumatoid arthritis (RA) and juvenile idiopathic arthritis (JIA). Additionally, the IL-1 receptor antagonist anakinra has demonstrated clinical effectiveness and is a promising therapeutic option for RA [38]. Ustekinumab, an IgG1 kappa monoclonal antibody, specifically targets the p40 subunit common to IL-12 and IL-23. By neutralizing this shared subunit, ustekinumab prevents IL-12 and IL-23 from interacting with their receptors on immune cells, which blocks subsequent pro-inflammatory signaling pathways, including Th1- and Th17-mediated pathways involved in the development of autoimmune and inflammatory diseases [48].

RORγt functions as a key regulator of Th17 cell differentiation and is a promising therapeutic target for autoimmune diseases. Inhibitors such as VTP-43472 and JTE-451 are currently being studied for their potential to modulate Th17 differentiation in the development of psoriasis [49].

The liver-microbiota axis is increasingly recognized as a key modulator of autoimmunity. In steroid-naive autoimmune hepatitis (AIH) patients, gut microbiota is characterized by reduced intraindividual diversity and altered taxonomic composition. Notably, increased abundance of Veillonella dispar correlates with disease severity, including elevated transaminases and histological activity, whereas decreased Bifidobacterium is associated with failure to achieve remission. These findings highlight the therapeutic potential of microbiota-targeted interventions, supporting the use of probiotics to maintain microbial homeostasis after remission or as adjunctive therapy in treatment-resistant AIH to reduce relapse and disease progression [52].

## 8. Conclusions

Evidence regarding IL-17’s role in AIH indicates a central function by promoting inflammatory cell infiltration, amplifying pro-inflammatory cytokine networks, and driving fibrogenic responses in key liver cell populations. Experimental data support the therapeutic potential of targeting the IL-17 axis. The balance between beneficial host defense and pathological immune activation remains incompletely understood, warranting further research into this immune pathway in autoimmune liver disease.

## Figures and Tables

**Table 1:** Summarization of the evidence regarding the role of IL-17 in the hepatic inflammatory response in AIH.

Authors	Year	Results
Zhao L et al. [36]	2011	Serum IL-17 levels and the number of IL-17–positive cells within hepatic inflammatory infiltrates were significantly elevated in patients with AIH compared with both healthy subjects and individuals with chronic HBV infection. Moreover, IL-17 was shown to enhance IL-6 production by hepatocytes, which, in turn, promotes further activation of Th17 cells.
Yu H et al. [39]	2010	Elevated IL-17 levels in the peripheral blood mononuclear cells of AIH patients, alongside hepatic infiltration by IL-17-positive cells in the animal models, strongly suggested that the IL-17 pathway plays a central role in AIH development. Treatment with a monoclonal antibody targeting IL-17A significantly reduced hepatic necrosis when compared with control groups.
Gutkowski K et al. [40]	2018	IL-6 and IL-17 levels were significantly elevated in patients with active AIH compared with those in remission and the control group. Serum IL-6 and IL-17 concentrations did not differ significantly between patients in remission and healthy controls. Negative correlation was observed between the severity of inflammatory lesions and serum IL-17 levels.
An J et al. [41]	2019	Serum levels of IL-17, IL-6, IL-21, and TNF-α were markedly elevated in patients with AIH compared with those in the control group. Moreover, serum IL-17 and TNF-α levels, as well as the frequency of Th17 cells, were positively correlated with serum transaminase levels.
Liang M et al. [42]	2018	The findings revealed significantly higher levels of IFN-γ, TNF-α, and IL-17A in patients with AIH and demonstrated that the pro-inflammatory Th1 cytokines (IFN-γ and TNF-α) together with IL-17A represent key mediators of Th1- and Th17-driven immune responses and may contribute to hepatic injury by promoting neutrophil infiltration.
He Q et al. [43]	2022	Their findings showed that γδ T cells from individuals with AIH exhibited significantly elevated levels of IL-17A and RORγt compared with healthy controls. Results indicate that TOX expression in γδ T cells is associated with Tγδ17 cell frequency, inflammatory status, and the clinical diagnosis of AIH.
Xia G et al. [44]	2018	The investigators observed elevated hepatic miR-155 expression, accompanied by increased levels of IL-17A, IL-23, and IL-10. Antagomir-155 attenuated liver injury in mice and diminished the aberrant upregulation of IL-17A and IL-23 in hepatic tissues. Moreover, administration of recombinant IL-17A (rIL-17A) resulted in significant increases in ALT, AST, and ALP levels, further highlighting IL-17’s contributory role in disease progression.
Buitrago-Molina LE et al. [45]	2021	The study identified the absence of erythropoietin along with marked up-regulation of caspase-3 (Casp3), IL-17, and IL-23R as key factors potentially contributing to the pronounced portal inflammation observed in the AIH-affected liver.
Huang J et al. [46]	2017	Using murine and human models, the researchers observed elevated levels of IL-17C and IL-17RE in liver tissues in both species, suggesting a potential role in disease development.
Grant CR et al. [47]	2014	Their findings indicate that CD39^+^ Tregs are reduced in number, exhibit impaired hydrolysis of pro-inflammatory nucleotides, and are less effective at suppressing IL-17 production. Furthermore, these CD39^+^ Tregs in AIH demonstrate a more pro-inflammatory phenotype, characterized by in-creased CD127 expression and a heightened capacity to produce IFN-γ and IL-17.
Yan S et al. [50]	2012	Reported a marked upregulation of IL-17 expression in hepatic tissues during Con A-induced hepatitis in murine models. Elevated IL-17 levels correlated with the severity of hepatic injury, as evidenced by increased ALT, histopathological findings, and increased secretion of TNF-α and IL-6.
Wu H et al. [53]	2021	In serum samples from AIH patients, levels of IL-17A, phosphorylated p38 (p-p38), and phosphorylated JNK (p-JNK) were elevated, whereas MKP-1 levels were reduced, relative to healthy controls. In the Con A-induced AIH model, p38 activation and MKP-1 suppression promoted the differentiation of CD4^+^ T cells into Th17 cells, thereby increasing IL-17A production.
El-Guindi M et al. [54]	2015	Serum IL-17 levels were significantly elevated in the AIH group compared to both the chronic liver disease and healthy control groups. Moreover, IL-17 levels were significantly higher in AIH patients without a response compared with those with a response to treatment.
Xu W et al. [55]	2023	Demonstrated that induced AIH mice exhibited significantly elevated ex-pression of inflammatory cytokines, including IFI16, IL-1β, IL-18, and IL-17, as well as markedly increased serum glutathione transaminase activity compared with controls. IL-17-mediated activation of STAT3 and subsequent transcriptional regulation of IFI16 were identified as key drivers of AIH-associated pyroptosis. Administration of an IL-17-neutralizing anti-body (nAb) in AIH mice resulted in substantial reductions in histological liver damage and serum aminotransferase levels.
Xiao Z et al. [56]	2024	Demonstrated that Lkb1 exerts differential control over the thymic development and peripheral maintenance of γδ T-cell subsets. Lkb1 deficiency compromised γδ T-cell viability, facilitated the expansion of IL-17-producing γδ T cells, and ultimately precipitated AIH, indicating the involvement of an additional IL-17-mediated mechanism.
Longhi MS et al. [47]	2012	The frequency of IL-17-producing cells, isolated directly from peripheral blood mononuclear cells, was significantly higher in patients than in healthy controls.

## Data Availability

No new data were created or analyzed in this study.

## Abbreviations

The following abbreviations are used in this manuscript:

AIH	Autoimmune hepatitis
ANA	Antinuclear antibodies
Anti-LC1	Antibodies against liver cytosol antigen type 1
Anti-LKM-1	Antibodies against liver–kidney microsomal type 1
APC	Antigen-presenting cells
ASC	Autoimmune sclerosing cholangitis
ASMA	Anti-smooth muscle antibodies
CMTM4	Chemokine-like MARVEL transmembrane domain-containing protein 4
CTL	Cytotoxic T lymphocytes
CTLA-4	Cytotoxic T-lymphocyte–associated antigen 4
DC	Dendritic cell
HCV	Hepatitis C virus
HLA	Human leukocyte antigen
HT	Hashimoto’s thyroiditis
IBD	Inflammatory bowel disease
IgG	Immunoglobulin G
IL-17	Interleukin-17
IL-17R	IL-17 receptor
ILC	Innate lymphoid cell
MAPK	Mitogen-activated protein kinase
MHC	Major histocompatibility complex
MS	Multiple sclerosis
NF-Kb	Nuclear factor-kB
NGF	Nerve growth factor
NK	Natural killer
NKT	Natural killer T
PDGF	Platelet-derived growth factor
RA	Rheumatoid arthritis
SARS-CoV-2	Severe acute respiratory syndrome coronavirus 2
SLE	Systemic lupus erythematosus
SNP	Single-nucleotide polymorphism
TCR	T cell receptor
TGF-β	Transforming growth factor beta
Th	T helper
TLR	Toll-like receptor
TNF	Tumor necrosis factor
TRAF	TNF receptor-associated factor
Treg	T regulatory
